# Raman spectroscopic sensing of carbonate intercalation in breast microcalcifications at stereotactic biopsy

**DOI:** 10.1038/srep09907

**Published:** 2015-04-30

**Authors:** R. Sathyavathi, Anushree Saha, Jaqueline S. Soares, Nicolas Spegazzini, Sasha McGee, Ramachandra Rao Dasari, Maryann Fitzmaurice, Ishan Barman

**Affiliations:** 1 Department of Pathology, School of Medicine, Case Western Reserve University, 10900 Euclid Avenue, Cleveland, OH 44106, USA; 2 Current Address: Sandor Lifesciences Pvt. Ltd, 8-2-326/5, Road No 3, Banjara Hills, Hyderabad-500034, India; 3 Departamento de Física, Universidade Federal de Ouro Preto, Ouro Preto, MG– 35400-000, Brazil; 4 Laser Biomedical Research Center, G. R. Harrison Spectroscopy Laboratory, Massachusetts Institute of Technology, 77 Massachusetts Avenue, Cambridge, MA 02139, USA; 5 Current Address: District of Columbia Department of Health, 899 North Capitol St NE, DC 20002, Washington; 6 Department of Mechanical Engineering, Johns Hopkins University, Baltimore, Maryland 21218, USA; 7 Department of Oncology, Johns Hopkins University, Baltimore, Maryland 21287, USA

## Abstract

Microcalcifications are an early mammographic sign of breast cancer and frequent target for stereotactic biopsy. Despite their indisputable value, microcalcifications, particularly of the type II variety that are comprised of calcium hydroxyapatite deposits, remain one of the least understood disease markers. Here we employed Raman spectroscopy to elucidate the relationship between pathogenicity of breast lesions in fresh biopsy cores and composition of type II microcalcifications. Using a chemometric model of chemical-morphological constituents, acquired Raman spectra were translated to characterize chemical makeup of the lesions. We find that increase in carbonate intercalation in the hydroxyapatite lattice can be reliably employed to differentiate benign from malignant lesions, with algorithms based only on carbonate and cytoplasmic protein content exhibiting excellent negative predictive value (93–98%). Our findings highlight the importance of calcium carbonate, an underrated constituent of microcalcifications, as a spectroscopic marker in breast pathology evaluation and pave the way for improved biopsy guidance.

Breast cancer is the most common malignant neoplasm and accounts for nearly a quarter of all cancers in women worldwide^1^. Spurred principally by mammography screening, an ever-increasing number of breast cancers are being detected before the development of axillary metastases^2^. However, while the increase in mammography screening has led to a dramatic increase in the detection of lesions, only 10–25% of mammographically suspicious lesions are subsequently determined to be malignant^3^,^4^. This results in unnecessary and costly biopsy procedures.

One of the primary features of diagnostic importance in mammography is the appearance of microcalcifications, with up to 50% of all non-palpable breast cancers being linked with this marker^5^. Despite their obvious importance in the diagnostic and prognostic landscape, microcalcifications remain poorly understood and may often confound clinical decision making. Moreover, mammography does not provide the requisite information to classify the two types of microcalcifications observed in breast tissue that have vastly different implications in terms of the underlying lesion pathology^6^. Type I microcalcifications consist of pure crystalline calcium oxalate dihydrate (weddellite crystals) and are seen almost exclusively in benign duct cysts and only rarely in non-invasive lobular carcinoma *in situ*^7^. These oxalate-type microcalcifications are most likely a product of sequestered duct secretions, and are therefore of little clinical concern. In contrast, type II microcalcifications are composed of calcium phosphates, mainly calcium hydroxyapatite with intercalated carbonate, deposited on a protein matrix and are associated with both benign and malignant proliferative lesions, including *in situ* and invasive ductal cancinoma^8^.

Owing to the clinical significance of microcalcifications, stereotactic breast needle biopsies are typically used to diagnose the mammographic lesion of interest. The complete diagnostic work up of such a process may require multiple interventional procedures leading to surging healthcare costs and a traumatic experience for the patient involved. Therefore, there is an urgent, unmet clinical need for a tool that can aid in targeted detection of type II microcalcifications and enable further differentiation between type II microcalcifications associated with benign lesions from those associated with malignancies. To address this need, our research has focused on the development of optical spectroscopy, especially Raman scattering, driven primarily by its exquisite molecular specificity and ability to perform real-time measurements in a minimally invasive, non-destructive manner. Raman scattering is an inelastic scattering process in which photons incident on a sample transfer energy to or from the vibrational and rotational modes of molecules in the sample. It is a two-photon process and can be thought of as the simultaneous absorption of an incident photon and emission of a Raman photon. The difference between the energies of these two photons corresponds to the transition of a molecule from one energy level to another. Because the energy levels are unique for each molecule, Raman spectra of tissue consist of multiple bands characteristic of specific molecular motions and thus provide a chemical fingerprint of the breast tissue. As a result, Raman spectroscopy and its vibrational counterpart, *i.e.* Fourier Transform Infrared (FTIR) spectroscopy, have been extensively used to investigate breast malignancies^9^,^10^,^11^,^12^. Critically, Raman spectroscopy is particularly sensitive to symmetric vibrations such as the symmetric phosphate vibrations in hydroxyapatite in tissue mineralization such as breast microcalcifications. Previous studies by us and others have highlighted the potential of Raman spectroscopy to detect microcalcifications associated with benign and malignant breast lesions^13^,^14^,^15^.

In the efforts towards developing Raman spectroscopy as a clinical tool for real-time guidance of stereotactic breast biopsies, important clues can be drawn from initial investigations of isolated microcalcifications performed in formalin-fixed paraffin-embedded thin tissue sections^13^. Our prior study demonstrated the ability of Raman spectroscopy to distinguish type II microcalcifications associated with benign breast lesions from those associated with breast cancer. This was the first time that type II microcalcifications associated with benign and malignant breast lesions had been distinguished other than by histopathology diagnosis at breast biopsy. This later distinction was made using principle component analysis (PCA), a widely used dimension reduction and data exploration method, which requires no *a priori* information regarding the chemical composition of the tissue structures under study. Furthermore, inspection of the Raman spectra and PCA vectors suggested that the hydroxyapatite calcifications formed in benign ducts contain smaller amounts of protein and larger amounts of calcium carbonate than those formed in malignant ducts. A more recent FTIR-based investigation by Stone and co-workers supports this conclusion and has also shown that the carbonate content of hydroxyapatite microcalcifications decreases with increasing lesion grade^16^.

All these studies, nonetheless, have been confined to fixed tissue section analyses using microspectroscopy, which represent an important first step but do not address the substantial complexity of real-time fiber probe-based measurements in tissue cores at stereotactic biopsy. While Raman spectroscopy can provide important structural information on the molecular composition of a cell or tissue sample, sample preparation can have a significant impact on the quantification and interpretation of spectra for their biochemical relevance. In particular, formalin fixing and paraffin embedding have been reported to affect a number of Raman features such as reduction of the amide-I peak (due to the formation of tertiary amides) and appearance of a new band at 1490 cm^−1^ 17. Additionally, there is lack of consensus with regard to a standard protocol for deparaffinization of paraffin embedded sections for Raman investigations, as each of the deparaffinization processes (including the one employed in our earlier study) presents its own challenges. Critically, fiber-probe based measurements interrogate significantly larger sampling volumes as compared to the mentioned microspectroscopy systems and the diagnostic impact of this on the ability to differentiate lesion types based on type II microcalcification composition is yet to be determined.

The aim of this study is, therefore, to develop and validate decision algorithms derived from Raman spectra acquired from freshly excised breast tissue cores using fiber-probes that are compatible with standard 9-gauge biopsy needles. The overarching aim of the decision algorithms is to identify spectral markers that can be used to differentiate the nature of the underlying breast lesion associated with type II hydroxyapatite microcalcifications *in vivo*.

## Results

Raman spectra were obtained from fresh breast biopsies from 26 patients undergoing stereotactic breast needle biopsy, using a compact Raman clinical system. Seventy-five of the tissue sites interrogated contained breast lesions with type II microcalcifications. Since the principal goal of the study was to examine the relationship between the chemical composition of hydroxyapatite microcalcifications and the nature of the underlying breast lesion, all further analysis was restricted to these tissue sites harboring such type II microcalcifications. Two were eliminated as outliers and provided poor model fits. The remaining 73 Raman spectra constituting this data set could be divided according to the histological assessment in the following manner: 42 tissue sites from 15 patients were diagnosed as fibrocystic change (FCC); 17 tissue sites from 6 patients diagnosed as fibroadenoma (FA); and 14 tissue sites from 5 patients diagnosed as invasive ductal carcinoma (IDC; n = 1) or ductal carcinoma *in situ* (DCIS; n = 13). FCC and fibroadenoma are among the most common benign conditions observed in the breast. FCC is an exaggerated response to hormonal changes during the menstrual cycle, manifested as fibrosis, adenosis or cyst formation^18^, whereas fibroadenoma is a benign tumor consisting of a fibroblastic stroma that contains elongated compressed ducts lined by benign appearing epithelium^19^.

### Calcium carbonate contribution to the Raman spectra of breast lesions with type II microcalcifications

**Figure 1:**
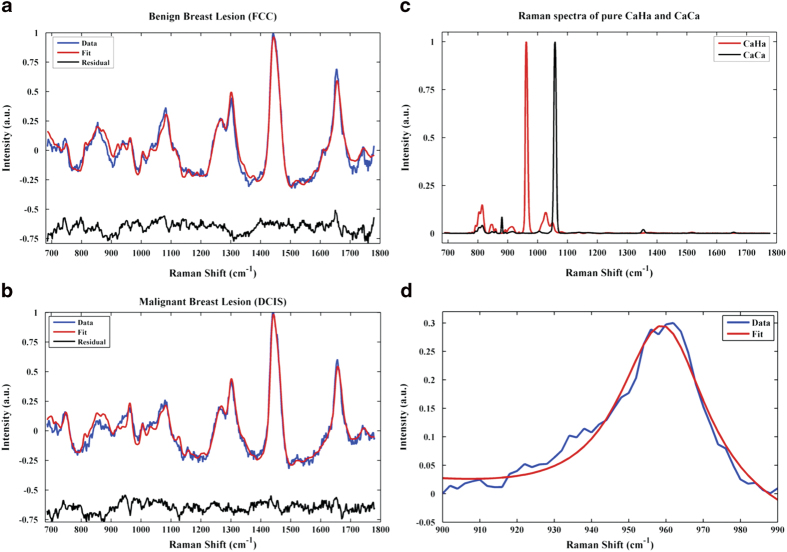
Representative Raman spectra of (**a**) a benign breast lesion (FCC) and (**b**) a malignant breast lesion (DCIS) with type II microcalcifications, with model fits and residuals; (**c**) Raman spectra of pure calcium hydroxyapatite (CaHa) and calcium carbonate (CaCa); and (**d**) 960 cm^−1^ Raman feature, characteristic of apatite structures, acquired from a benign lesion with type II microcalcifications. The overlaid Lorentzian fit is used to compute the FWHM of the band and, thus, to provide a measure of the carbonate intercalation in the apatite structure.

Figure 1a,b show typical Raman spectra of benign (FCC) and malignant (DCIS) breast lesions with type II microcalcifications, respectively, with the corresponding model fits and residuals. A prominent symmetric phosphate stretching mode band at 960 cm^−1^ is present in both of these Raman spectra, confirming the presence of type II hydroxyapatite microcalcifications. As detailed in our prior investigations on isolated microcalcifications, the Raman spectrum of pure stoichiometric calcium hydroxyapatite consists of four phosphate vibrational modes, two of which are outside the considered spectral range. The mentioned peak is attributed to the *v*_1_(PO_4_) mode of the “free” tetrahedral phosphate ion. However, in comparison to the sharp phosphate band at 960 cm^−1^ observed in the Raman spectrum of pure calcium hydroxyapatite (Fig. 1c), there is significant broadening of this feature in the spectra acquired from both benign and malignant breast lesions with type II microcalcifications.

Since broadening of this peak has been associated with the concomitant presence of calcium carbonate, further analyses were carried out to elucidate the impact of such carbonated apatite models on the diagnosis of breast lesions associated with microcalcifications. Broadening of the 960 cm^−1^ band was assessed quantitatively by measuring the full width at half maximum (FWHM) using an automated curve fitting algorithm that matches a Lorentzian profile with the experimental data. Table 1 shows the mean FWHM of the 960 cm^−1^ band (Fig. 1d) for the FCC, FA and DCIS/IDC groups. These results show a roughly 1.5-fold increase in the mean FWHM of the 960 cm^−1^ band for the benign breast lesion groups (FCC = 48.71; FA = 47.05) when compared with that for the malignant breast lesion group (DCIS/IDC = 33.14). We believe that this broadening of the hydroxypatite phosphate band is due to intercalation of carbonate ions into the apatite lattice, which reduces the symmetry of its unit cell^20^,^21^. The peak broadening results from a loss of long-range translational order in the apatite structure, and increases as the carbonate content of the sample increases. It is worth noting that even though our custom-built fiber probe interrogates larger volumes consistent with the sampled tissue in standard 9-gauge biopsy needles, it has sufficient sensitivity to report on the differences between the lesions based on the carbonate intercalation, an underrated marker for breast pathology.

The presence of calcium carbonate is further evidenced by the presence of a smaller band at *ca*. 1072 cm^−1^ in the Raman spectra of benign and malignant breast lesions with type II microcalcifications (Fig. 1a,b), which can be attributed to calcium carbonate *v*_1_(CO_3_) mode. The amount of calcium carbonate present was assessed quantitatively by determining the relative concentration of 9 Raman-active constituents of breast tissue (calcium carbonate, calcium hydroxyapatite, calcium oxalate, epithelial cell nuclei and cytoplasm, stromal collagen fibers, fat cells, cholesterol-like extracellular deposits and beta-carotene) based on ordinary least squares modelling. Using this model, significant Raman fit coefficients (FC) for calcium hydroxyapatite, calcium carbonate and/or calcium oxalate were observed in all breast lesions harboring microcalcifications. The mean and standard deviation for the calcium carbonate fit coefficient (FC) for the FA, FCC and DCIS/IDC groups is provided in Table 1. These results again show a substantive increase in the mean calcium carbonate FC for the benign breast lesion groups (FCC = 5.13; FA = 3.60) when compared with that for the malignant breast lesion group (DCIS/IDC = 2.74) Employing both the FWHM of the 960 cm^−1^ peak and the calcium carbonate FC, statistically significant differences were observed between the benign and malignant groups (p < 0.05). The significance values were calculated based on the Wilcoxon rank-sum test, which does not assume a normal distribution of data. Expectedly, a positive correlation between broadening of the 960 cm^−1^ phosphate band (FWHM) and the calcium carbonate content (FC) of the tissue site was also noted (r = 0.68). The slight discrepancy in the trends of computed FWHM and calcium carbonate FC values can be attributed to the different modeling approaches, namely Lorentzian fitting of the 960 cm^−1^ peak and ordinary least squares fitting of the spectral profile, and the inherent sources of error in each case. Due to the presence of only one IDC sample in the fresh tissue core dataset, it was not possible to determine the possibility of using calcification parameters to differentiate between invasive and *in situ* carcinomas; but findings from Baker *et al.* point toward the presence of a progressive decrease in carbonate content from benign to increasingly malignant breast lesions^13^.

Table 2 shows the mean values and the standard deviations of the calcium carbonate to calcium phosphate FC ratios for the DCIS/IDC, FA and FCC groups. Here again the variations between the FCC and DCIS/IDC groups are evident with a nearly 2-fold increase in the calcium carbonate intercalation in the apatite structures for the former benign lesions. However, for the fibroadenoma tumors the ratios of carbonate to phosphate appear to be in the same range as that of the malignant group. Thus, this parameter proved to be less useful than the FWHM of the 960 cm^−1^ feature and the FC of calcium carbonate in parameter-based decision algorithms developed for the diagnosis of breast, described below.

Taken together, these results confirm our earlier inference that broadening of the 960 cm^−1^ phosphate band in the Raman spectra of isolated type II microcalcifications is due to intercalated calcium carbonate^13^, and show that this significant spectral finding can still be detected in the Raman spectra of tissue cores from breast lesions containing type II microcalcifications.

### Protein contribution to the Raman spectra of breast lesions with type II microcalcifications

The two major proteins present in breast tissue are actin, the predominant protein in the cytoplasm of ductal and lobular epithelial cells^22^, and collagen, the predominant protein in the extracellular stroma^23^, which is also present in the matrix of type II microcalcifications^23^. The contributions of these proteins to the Raman spectra of benign and malignant lesions with type II microcalcifications is not as easily seen on visual inspection as those of calcium hydroxyapatite and carbonate. However, the amount of these proteins present was assessed quantitatively by modeling of the Raman spectra. Table 3 shows the mean collagen and epithelial cell cytoplasm (actin) FC for the FA, FCC and DCIS/IDC groups. These results show a roughly 1.5-fold increase in the mean collagen FC for the benign breast lesion groups (FCC = 31.11; FA = 26.20) when compared with that for the malignant breast lesion group (DCIS/IDC = 18.57). This is a similar trend to that seen with the calcium hydroxyapatite FWHM and calcium carbonate FC.

Our prior observations^13^ and those by other investigators^16^ in microspectroscopy studies of isolated type II microcalcifications showed an increase in protein, presumed to be matrix collagen, in type II microcalcifications associated with malignant breast lesions. The current result for the collagen FC in fresh breast biopsy tissue cores is, however, not surprising. This is because most collagen in benign or malignant breast lesions is present in the stroma and not in the microcalcifications, which again represent only a small fraction of the tissue. In contrast, we did observe a roughly 3- to 6-fold increase in the FC of epithelial cell cytoplasm (actin) in malignant lesions (DCIS/IDC = 12.36) when compared to that in benign breast lesions (FCC = 4.67; FA = 1.86) with type II microcalcifications, shown in Table 2. This is not surprising either, as DCIS and IDC are both proliferative lesions with increases in both the size and population density of malignant ductal epithelial cells^23^. Therefore ductal epithelial cells represent a larger fraction of the tissue in these malignant breast lesions than in benign breast lesions. To the best of our knowledge, this is the first spectroscopic study that links the presence of microcalcifications with the protein concentration of the ductal epithelial cells.

### Calcium carbonate and protein-based empirical decision algorithms

**Figure 2:**
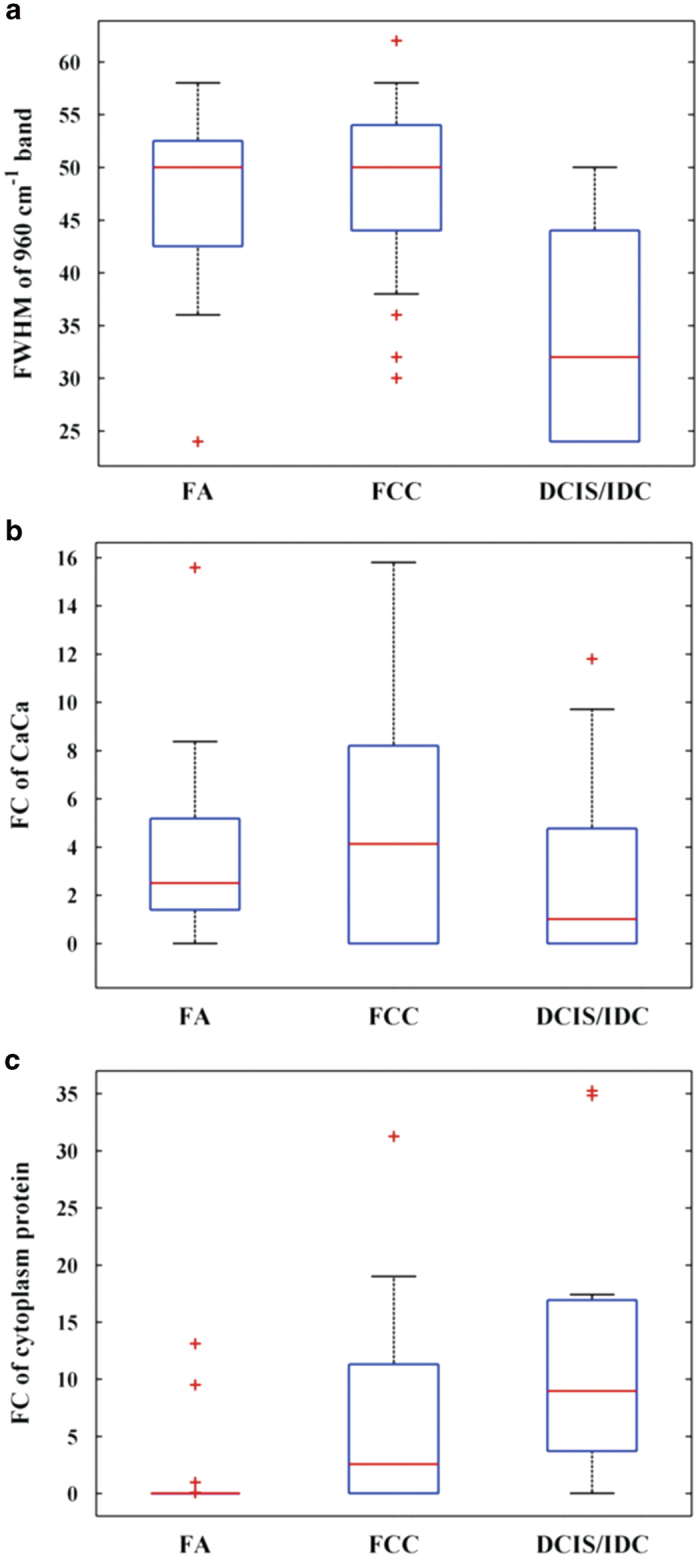
Box plots summarizing the Raman spectra-derived data for the different breast lesions with type II microcalcifications: (**a**) the FWHM of the 960 cm^−1^ band, (**b**) the FC of calcium carbonate (CaCa) and (**c**) the FC of cytoplasmic protein content.

In order to exploit the differences seen in calcium carbonate and cytoplasmic protein (actin)^22^, we sought to develop parameter-based decision algorithms to distinguish malignant from benign lesions with type II microcalcifications. To do this, we first assessed the likely diagnostic value of Raman spectral measurements of all 9 breast tissue model components using box plots. Box plots graphically depict groups of numerical data through their quartiles and help display differences between populations or samples without making any assumptions of the underlying statistical distribution. Here, the lower limit and upper limit of the box represent the first and third quartiles, and the center line represents the second quartile (median). From the box plots shown in Fig. 2, the benign (FCC/FA) and malignant breast lesions (DCIS/IDC) with type II microcalcifications show sufficient differentiation based on both the FWHM value of the 960 cm^−1^ band and the FC of epithelial cell cytoplasm. The groups show reasonable separation based on the calcium carbonate FC as well, though the overlap appears to be slightly greater than in the other cases. Indeed, the calcium carbonate FC and the FWHM value of the 960 cm^−1^ peak both provide quantitative measures of the carbonate intercalation in the apatite structures. Nevertheless, in this particular dataset, the FWHM value appears to provide better discrimination, which could likely be a result of the different modelling approaches used to arrive at these two measures.

Based on the results of the box plot analysis, we developed several diagnostic algorithms. In developing these algorithms, we considered the positive class to be cancer and the other classes, fibroadenoma and fibrocystic change, to be negative. The performance of the algorithms was assessed using the following metrics: sensitivity (SE), specificity (SP), positive predictive value (PPV), negative predictive value (NPV) and overall accuracy (OA), based on the true positive (TP), false positive (FP), true negative (TN) and false negative (FN) results, per the standard definitions given below: SE = TP/(TP + FN); SP = TN/(TN + FP); PPV = TP/(TP + FP); and NPV = TN/(TN + FN). We first devised two single-parameter Raman spectral algorithms to distinguish benign and malignant lesions with type II microcalcifications, one based on the FWHM of the 960 cm^−1^ band and the other on the FC of epithelial cell cytoplasm. The optimal threshold values for the single parameter decision algorithms were set empirically to maximize the number of correct diagnoses of malignant breast lesions. Using an optimal decision threshold of 46, the 960 cm^−1^ band FWHM algorithm yielded a negative predictive value (NPV) of 98%, with a sensitivity (SE) of 93%, specificity (SP) of 75%, positive predictive value (PPV) of 46% and overall accuracy of 77%, for the distinction of malignant breast lesions (DCIS + IDC) from benign lesions (FCC + FA) with type II microcalcifications. In comparison, using an optimal threshold of FC = 9, the epithelial cell cytoplasm algorithm yielded a slightly decreased NPV of 93% and SE of 71%, but a slightly increased SP of 82%, PPV of 48% and overall accuracy of 80%.

**Figure 3:**
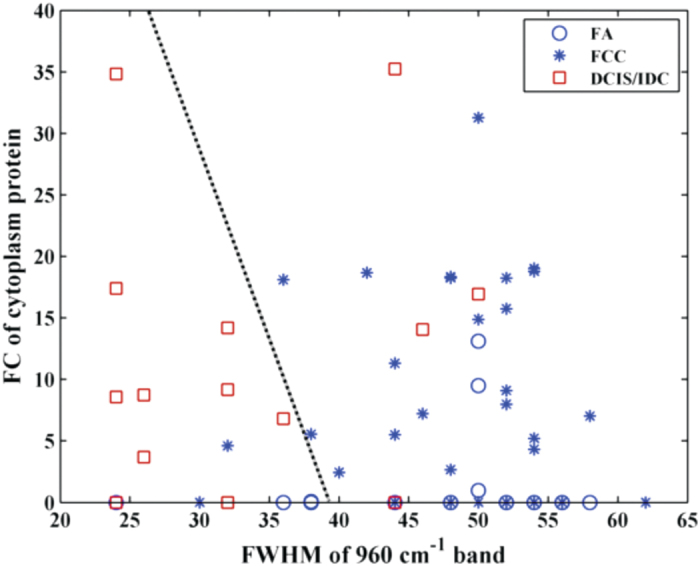
Two-parameter Raman decision algorithm developed to distinguish malignant from benign breast lesions with type II microcalcifications. DCIS/IDC sites are depicted by red squares, while the FCC and FA lesions are marked by blue stars and blue circles, respectively.

To further improve diagnostic performance, we devised a 2-parameter decision algorithm using both the FWHM of the 960 cm^−1^ band and the FC of epithelial cell cytoplasm, shown in Fig. 3. Using a logistic regression-derived algorithm that optimizes the number of correct diagnoses of malignant breast lesions, the decision model yielded the same NPV as the single parameter epithelial cell cytoplasm algorithm (93%), but a further increase in SP to 85%, PPV to 53% and overall accuracy to 83%, for the distinction of malignant breast lesions (DCIS + IDC) from benign lesions (FCC + FA) with type II microcalcifications. The NPV of the empirical decision algorithms using fresh tissue measurements from breast lesions with type II microcalcifications is comparable to that achieved in our study of isolated type II microcalcifications using PCA (97%)^13^. Table 4 shows a side-by-side comparison of the performance of the decision algorithms developed in this study.

## Discussion

Raman spectroscopy is well suited to the study of breast lesions with microcalcifications, as it provides a definitive chemical analysis of the lesional tissue and any microcalcifications it contains^13^,^24^,^25^,^26^,^27^,^28^,^29^,^30^,^31^. It has been used successfully to study other tissues with hydroxyapatite-based mineralization such as bone^32^,^33^. The bone studies have shown that the symmetric phosphate vibrations in hydroxyapatite in tissue mineralization are sensitive to both the tissue microenvironment and ionic (in this case carbonate) intercalation into the hydroxyapatite matrix^13^. In particular, if the apatite lattice is disordered and/or amorphous, the phosphate symmetric stretch band is typically seen at 945–950 cm^−1^. If the apatite lattice is carbonate substituted, the phosphate symmetric stretch band is typically seen at 955–960 cm^−1^. For non-substituted crystalline apatite, the phosphate symmetric stretch band is typically seen at 962–964 cm^−1^. Further, for apatites with a carbonate substituted lattice, the phosphate symmetric stretch band around 960 cm^−1^ is broadened as also evidenced from our findings here^14^,^15^,^16^,^34^.

Breast microcalcifications are inherently complex materials that may be composed of various amounts of all three apatite species. However, the predominant apatite species in type II microcalcifications is the carbonate substituted apatite structures, as the dominant phosphate symmetric stretch band in breast lesions in type II microcalcifications was seen at ~960 cm^−1^ in this study of fresh breast biopsy tissue cores and our previous study of formalin-fixed breast tissue sections^13^. At this time, the specific mechanism for hydroxyapatite formation and, more importantly, its role in breast tumor progression is not fully understood, although it is likely that the latter stems from several interconnected effects including direct enhancement of mitogenesis of mammary cells, upregulation of matrix metalloproteinases (MMPs) and increased expression of prostaglandin and inflammatory cytokines^35^.

**Figure 4:**
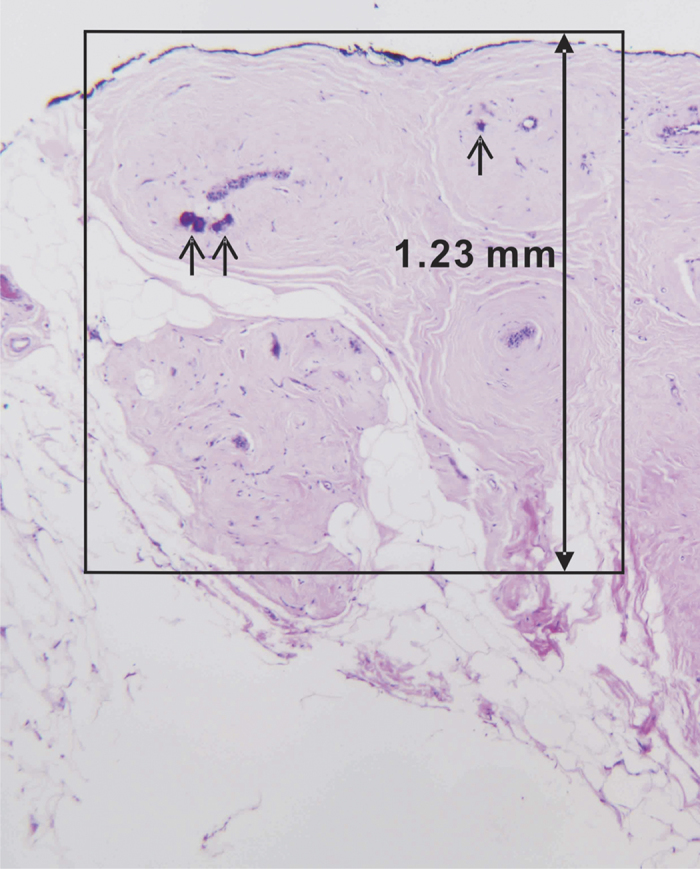
Photomicrograph of an H&E-stained breast biopsy tissue core harboring type II microcalcifications within a benign breast lesion (fibrocystic change)(4X). The box highlights the lesion interrogation volume. The microcalcifications (arrows) occupy only a small fraction of the overall tissue volume sampled by the Raman spectroscopy fiber-probe.

In this study, we also observed a roughly 1.5-fold increase in calcium carbonate, as measured by the calcium carbonate model FC, in benign breast lesions (FCC/FA) as compared to malignant breast lesions (DCIS/IDC). Thus, in this study, we confirmed by quantitative measurements in fresh breast biopsy tissue cores what we had only inferred from our previous observations in isolated microcalcifications in fixed tissue sections. Furthermore, the presence of greater carbonate content in relatively benign lesions reinforces the idea that purely apatite structures are deposited by tumourigenic cells thereby indicating specific mineralization potential may be associated with cell phenotype^36^. This shows that these carbonate-intercalation based spectral findings (first observed in isolated microcalcifications) can still be detected in the Raman spectra of breast lesions containing type II microcalcifications, even though microcalcifications represent only a small fraction of the tissue volume interrogated (Fig. 4).

In this study, we also observed an increase in collagen, as measured by the collagen FC, in benign breast lesions when compared with malignant breast lesions, which reflects stromal proliferation in benign breast lesions. We also observed for the first time an increase in actin, measured as the FC of epithelial cell cytoplasm, in malignant breast lesions when compared to that in benign breast lesions with type II microcalcifications. This observation is along expected lines as ductal epithelial cells represent a larger fraction of the tissue in malignant than in benign breast lesions.

Based on these results, we developed Raman decision algorithms to distinguish benign and malignant breast lesions with type II microcalcifications based on their calcium carbonate and protein (actin) content^24^. We observed that the overall performance was best for a 2-parameter empirical algorithm, which had a NPV of 95% and PPV of 61%. However, the best NPV (98%) was achieved, at the expense of a slightly worse PPV (46%), using a single parameter empirical algorithm based only on the FWHM of the 960 cm^−1^ feature. This single parameter algorithm has the potential advantage that it does not require spectral modelling (either employing PCA or our breast model), so data analysis could be faster making it easier to implement clinically than a model-based algorithm.

### Conclusion and future directions

This pilot study represents the first demonstration of differentiation of benign and malignant breast lesions using the carbonate intercalation signature in type II microcalcifications in freshly excised tissue. Specifically, we show that the high specificity of the acquired Raman spectra allows differentiation between the underlying lesions harboring the microcalcifications with NPV of 93–98%. The data recorded in the present study also exhibits good agreement with our previous findings in formalin-fixed, deparaffinized thin tissue sections thereby confirming that that calcium carbonate, a poorly understood and relatively unexplored constituent of breast microcalcifications, forms an important molecular marker – clinical translation of which can assist in defining cancer onset/risk. Studies with a larger patient pool are currently under way at Johns Hopkins Medical Institutions to further expand and validate our diagnostic algorithm in this and other clinical settings. The precise merits of each parameter proposed here (FWHM of the 960 cm^−1^ peak, FC of calcium carbonate and carbonate to phosphate ratio) will also be re-evaluated through a prospective prediction on a larger cohort of patients. In particular, we would like to ascertain *in vivo* the linkage between chemical composition of microcalcifications and pathology grade/sub-grade of the associated lesions, as progression to invasion is commonly related to tumor grade. Ultimately, we envision that the microcalcification information can be coupled to recognized breast cancer markers, prominently the dynamic epithelial-stromal molecular “interactome”, to provide a comprehensive index for clinical diagnosis and prognostication.

Based on the promising results of this pilot study, we envision that this method can be readily translated to serve as a real-time guidance mechanism for stereotactic breast biopsies, improving microcalcification retrieval and, as a result, enhancing the diagnostic yield. The upshot of such an approach would be to significantly reduce the excision of tissue cores that do not harbor clinically relevant lesions, despite harboring microcalcifications. Additionally, recent investigations suggest that microcalcifications may also predict more aggressive tumor behavior and poorer clinical outcomes^37^,^38^,^39^. The value-added information provided by Raman spectroscopy could, therefore, also inform triaging of the small (~1–2 mm diameter) tissue cores for ancillary studies, routinely performed on breast cancer specimens to determine patient treatment strategy.

## Methods

### Patient population

Breast tissue was obtained from 26 patients (all female; ages 38–79) undergoing vacuum assisted stereotactic core needle breast biopsy procedures in the Breast Health Center at University Hospitals-Case Medical Center. All studies were approved by the Case Cancer Institutional Review Board and the Massachusetts Institute of Technology Committee on the Use of Humans as Experimental Subjects. Informed consent was obtained from all subjects prior to their biopsy procedures. All experiments were performed in accordance with the approved guidelines and regulations.

### Raman spectroscopy system

The portable clinical Raman spectroscopy system (instrument and optical fiber-probe based in these studies was developed at the Laser Biomedical Research Center at the Massachusetts Institute of Technology (MIT), previously described in detail^40^. The instrument uses an 830 nm InGaAs diode laser (Process Instruments, Salt Lake City, UT) as an excitation source to deliver light to the tissue *via* a custom-designed optical fiber probe. The probe consists of a single excitation fiber surrounded by nine collection fibers. The excitation fiber terminates in a short-pass filter centered at 830 nm that transmits the laser excitation light while blocking the fiber background (*i.e.* the Raman and fluorescence signal generated by the excitation fiber). The distal end of the probe has a sapphire ball lens that focuses the excitation light onto the tissue and collects the scattered light from the tissue into the nine collection fibers. The light on the collection path traverses through a long pass filter that suppresses the elastically scattered light emanating from the tissue to avoid further generation of a probe background. The linear array of collection fibers at the proximal end of the fiber is coupled to a spectrograph for dispersion onto a back illuminated, deep depletion, thermoelectrically-cooled CCD detector (PIXIS 256, 26 × 26 μm pixels, 1024 × 256 array, Princeton Instruments). The laser power at the sample (freshly excised tissue cores) was maintained in the range of 98 to 105 mW. The Raman spectra were acquired by vertical binning prior to averaging 10 successive frames, each with an acquisition time of 0.25 s, for a total collection time of 2.5 s. The estimated tissue interrogation volume using our fiber-probe is 1.23 mm^3^ driven by the functional requirement of detecting the presence of lesions in tissue cores with *ca.* 2 mm diameter^41^. While desirable for real-time biopsy guidance, the substantially larger interrogation volume may give rise to detection sensitivity issues, not observed in previous studies employing microspectroscopy systems. Understanding and characterizing this trade-off is one of the focal points of the current investigation.

### Data collection

All data were obtained *ex vivo* from freshly excised biopsy specimens in the Breast Health Center within 30 minutes of excision. The tissue cores collected during the needle biopsy procedure were placed inside a Petri dish moistened with saline solution. Individual tissue cores of interest were then selected and placed sequentially on an aluminum block, which was in turn positioned inside a light-tight black box for data collection. Spectra were collected from several tissue sites of interest on each core (typically normal tissue, lesions (grossly abnormal tissue) without microcalcifications and lesions with microcalcifications) identified by gross inspection and in comparison with the specimen radiograph. Spectra were also collected from different cores in each biopsy and thus the number of spectra varied from patient to patient. Real time data analysis was used to help confirm data collection from tissue sites with microcalcifications.

Following data collection, the tissue sites from which Raman spectra were obtained were marked with multicolored colloidal inks to uniquely identify each tissue site. The tissue was then fixed in 10% neutral buffered formalin, routinely processed, paraffin embedded, cut into tissue sections and stained with hematoxylin and eosin (H&E). The H&E stained tissue sections were then examined by an experienced breast pathologist, who was blinded to the results of the Raman analysis. The colored ink spots marking the sites interrogated spectroscopically were visible on the H&E stained tissue sections, and were used for comparison of histopathology and spectroscopy results. In our study, breast lesions were classified as harboring microcalcifications if microcalcifications were seen at that tissue site either on the specimen radiograph or on the H&E stained tissue sections.

### Data analysis

The instrument was calibrated daily prior to data collection. Wave number calibration was performed using a spectrum from 4-acetamidophenol (*Tylenol*^*©*^) with known Raman peak positions. To correct the Raman spectra for the system wavelength response, a spectrum from a wavelength-calibrated tungsten halogen lamp diffusely scattered from a reflectance standard, barium sulphate (BaSO4), was used. Additionally, to eliminate the probe background, spectra were collected from a roughened aluminium surface. Different weightings of the aluminum spectra were subtracted with the optimal one chosen based on the tissue optical properties. Contributions from cosmic rays were removed using a derivative filter. Finally, to remove the background fluorescence, the spectra were least squares fitted with sixth order polynomials, which were then subtracted from the raw spectra before further analysis. The data set was normalized to the most intense peak (1445 cm^−1^) to remove any possible intensity biases.

The calibrated, background subtracted and normalized Raman spectra were then fit with a previously developed breast model^24^, in which the Raman tissue spectrum is considered as a linear combination of its constituent basis spectra, *P*_*i*_, and their concentrations, *C*_*i*_, at wave number *ω*, equation (1).


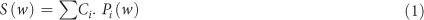


In this study, the model includes basis spectra obtained from: epithelial cell nuclei, epithelial cell cytoplasm, fat cells, cholesterol-like extracellular deposits, beta-carotene, stromal collagen fibers, calcium oxalate (synthesized), reagent grade calcium hydroxyapatite (Sigma, St. Louis, MO, USA) and calcium carbonate (Sigma, St. Louis, MO, USA). The goal of the multivariate analysis was to predict the chemical composition of the interrogated tissue and in the process to determine chemical markers that could help indicate changes in function of the complex tissue matrix (*i.e.* presence of malignancy). Therefore, ordinary least squares fitting was used to find the contribution of the spectra of the constituents of the basis model to each of the observed spectra by minimizing the sum of squared residuals, where the residual represents the difference between the acquired spectrum and the best fit value. This yields fit coefficients (FC) that are quantitative measures of the spectral contributions from the basis spectra thereby providing information about the morphological and biochemical composition of the tissue. The goodness of the fit was estimated qualitatively by visual inspection of the residual (measured spectrum minus model fit) and quantitatively by calculation of the standard deviation of the residual. In this regard, a Raman spectrum was eliminated from ensuing analysis if the standard deviation of the residual was observed to be greater than 0.2 or if it was determined to be an outlier using the Student’s t-test employing a Mahalanobis distance function.

## Author Contributions

R.S., A.S., R.R.D., M.A.F., I.B. designed the research; R.S., A.S., S.M. acquired spectroscopic data from biopsy tissue cores; M.A.F. performed histopathological evaluation of the tissue specimen; R.S., J.S.S., N.S., I.B. analyzed the data; R.S., M.A.F. and I.B. wrote the manuscript with help of all the authors.

## Additional Information

**How to cite this article**: *Sci. Rep.* **5**, 9907; doi: 10.1038/srep09907 (2015).

## Figures and Tables

**Table 1:** Mean values of the FWHM of 960 cm^−1^ peak and the calcium carbonate FC in benign and malignant breast lesions with microcalcifications.

**Lesion with Microcalcifications**	**FWHM 960 cm**^**−1**^**(Mean ± SD)**	**Calcium Carbonate FC (Mean ± SD)**
DCIS/IDC	33.14 ± 9.34	2.74 ± 3.71
FA	47.05 ± 8.78 (p = 0.0065)	3.60 ± 3.88 (p = 0.0001)
FCC	48.71 ± 6.90 (p = 0.015)	5.13 ± 4.98 (p = 0.0025)

**Table 2:** Ratio of calcium carbonate to calcium phosphate FC in benign and malignant breast lesions with microcalcifications.

**Lesion with Microcalcifications**	**CaCa / CaHa Ratio**
DCIS/IDC	0.34 ± 0.57
FA	0.35 ± 0.46
FCC	0.76 ± 1.04

**Table 3:** Mean values of collagen and epithelial cell cytoplasm FC in benign and malignant breast lesions with microcalcifications.

**Lesion with Microcalcifications**	**Collagen FC (Mean ± SD)**	**Epithelial Cell Cytoplasm FC (Mean ± SD )**
DCIS/IDC	18.80 ± 13.19	12.12 ± 11.13
FA	25.88 ± 12.97 (p = 0.002)	1.39 ± 3.79 (p = 0.01)
FCC	31.03 ± 17.97 (p = 0.004)	6.44 ± 8.14 (p = 0.007)

**Table 4:** Comparison of performance of the empirical decision algorithms to distinguish benign from malignant breast lesions with type II microcalcifications.

**Empirical Decision Algorithm**	**SE**	**SP**	**PPV**	**NPV**	**OA**
FWHM of 960 cm^−1^ peak alone (threshold value = 46)	93%	75%	46%	98%	77%
FC of cytoplasm protein alone (threshold value = 9)	71%	82%	48%	93%	80%
FWHM of 960 cm^−1^ peak vs. FC of cytoplasm protein	71%	85%	53%	93%	83%
